# Atlanta Society of Medicine

**Published:** 1891-01

**Authors:** 


### ATLANTA SOCIETY OF MEDICINE.

At last meeting the following officers were elected for this year:

President—Dr. F. W. McRae.
Vice-President—Dr. H. G. Hobbs.
Secretary—Dr. J. H. Childs.
Treasurer—Dr. E. Van Goidtsnoven.

With this able set of officers we predict a bright future for the Society.

---

We desire to call our readers' attention to our advertising department.

